# When Paying Attention Pays Back: Missense Mutation c.1006G>A p. (Val336Ile) in *PRKAG2* Gene Causing Left Ventricular Hypertrophy and Conduction Abnormalities in a Caucasian Patient: Case Report and Literature Review

**DOI:** 10.3390/ijms25179171

**Published:** 2024-08-23

**Authors:** Emanuele Micaglio, Lara Tondi, Sara Benedetti, Maria Alessandra Schiavo, Antonia Camporeale, Giandomenico Disabato, Andrea Attanasio, Gianluigi Guida, Gianpaolo Carrafiello, Massimo Piepoli, Pietro Spagnolo, Carlo Pappone, Massimo Lombardi

**Affiliations:** 1 Arrhythmology Department, IRCCS Policlinico San Donato, Piazza E. Malan, San Donato Milanese, 20097 Milan, Italy; emanuele.micaglio@grupposandonato.it (E.M.); carlo.pappone@grupposandonato.it (C.P.); 2 Institute for Molecular and Translational Cardiology (IMTC), IRCCS Policlinico San Donato, Piazza E. Malan, San Donato Milanese, 20097 Milan, Italy; 3 Multimodality Cardiac Imaging Section, IRCCS Policlinico San Donato, Piazza E. Malan, San Donato Milanese, 20097 Milan, Italygiandomenico.disabato@grupposandonato.it (G.D.); massimo.lombardi@grupposandonato.it (M.L.); 4 Postgraduate School in Radiodiagnostics, Università degli Studi di Milano, 20122 Milan, Italy; 5 Cardiology Unit IRCCS Azienda, Ospedaliero-Universitaria di Bologna, 40138 Bologna, Italy; 6 Department of Experimental, Diagnostic and Specialty Medicine University of Bologna, 40138 Bologna, Italy; 7 Clinical Cardiology, IRCCS Policlinico San Donato, Piazza E. Malan, San Donato Milanese, 20097 Milan, Italy; 8 Department of Diagnostic and Interventional Radiology, Foundation IRCCS Ca’ Granda-Ospedale Maggiore Policlinico, Via Francesco Sforza 35, 20122 Milan, Italy; 9 Department of Biomedical Sciences for Health, University of Milan, Via Festa del Perdono 7, 20122 Milan, Italy; 10 Unit of Radiology, IRCCS Policlinico San Donato, Piazza E. Malan, San Donato Milanese, 20097 Milan, Italy; 11 Department of Cardiology, Vita-Salute San Raffaele University, 20132 Milan, Italy

**Keywords:** left ventricular hypertrophy, hypertrophic cardiomyopathy, *PRKAG2* cardiomyopathy, cardiac magnetic resonance, genetic test

## Abstract

*PRKAG2* cardiomyopathy is a rare genetic disorder that manifests early in life with an autosomal dominant inheritance pattern. It harbors left ventricular hypertrophy (LVH), ventricular pre-excitation and progressively worsening conduction system defects. Its estimated prevalence among patients with LVH ranges from 0.23 to about 1%, but it is likely an underdiagnosed condition. We report the association of the *PRKAG2* missense variant c.1006G>A p. (Val336Ile) with LVH, conduction abnormalities (short PR interval and incomplete right bundle branch bock) and early-onset arterial hypertension (AH) in a 44-year-old Caucasian patient. While cardiac magnetic resonance (CMR) showed a mild hypertrophic phenotype with maximal wall thickness of 17 mm in absence of tissue alterations, the electric phenotype was relevant including brady–tachy syndrome and recurrent syncope. The same variant has been detected in the patient’s sister and daughter, with LVH + early-onset AH and electrocardiographic (ECG) alterations + lipothymic episodes, respectively. Paying close attention to the coexistence of LVH and ECG alterations in the proband has been helpful in directing genetic tests to exclude primary cardiomyopathy. Hence, identifying the genetic basis in the patient allowed for familial screening as well as a proper follow-up and therapeutic management of the affected members. A review of the *PRKAG2* cardiomyopathy literature is provided alongside the case report.

## 1. Introduction

Hypertrophic cardiomyopathy (HCM) is a cardiac disorder characterized by hypertrophy of the left ventricular (LV) wall. The clinical presentation of HCM is highly variable and often associated with atrial and ventricular arrhythmias, ischemia and eventually heart failure. However, many patients are asymptomatic and diagnosed during routine examinations.

To date, several genes have been associated with HCM, mostly with autosomal dominant inheritance [1]; however, mutations in eight sarcomere genes (*MYH7*, *MYBPC3*, *TNNI3*, *TNNT2*, *TPM1*, *MYL2*, *MYL3* and *ACTC1*) can diagnose 60% of familial HCM cases [2].

HCM can be isolated or associated with syndromic conditions, such as Danon disease, Fabry disease, Noonan syndrome and *PRKAG2* syndrome (PRS). PRS is a rare, early-onset, autosomal dominant (AD) phenotype associated with mutations in the *PRKAG2* gene (OMIM *602743), encoding the non-catalytic gamma subunit of the AMP-activated protein kinase. This disorder includes LV hypertrophy (LVH), ventricular pre-excitation and progressive conduction system abnormalities. Among patients with suspected HCM, the estimated prevalence of PRS ranges from 0.23 to about 1% [3], but it is likely an underdiagnosed condition. Indeed, its prevalence rises to 29% in patients with LVH and pre-excitation, and prevalence estimation might be inaccurate since LVH and pre-excitation do not always coexist [4,5].

## 2. Case Report

### 2.1. Case Presentation

**Figure 1:**
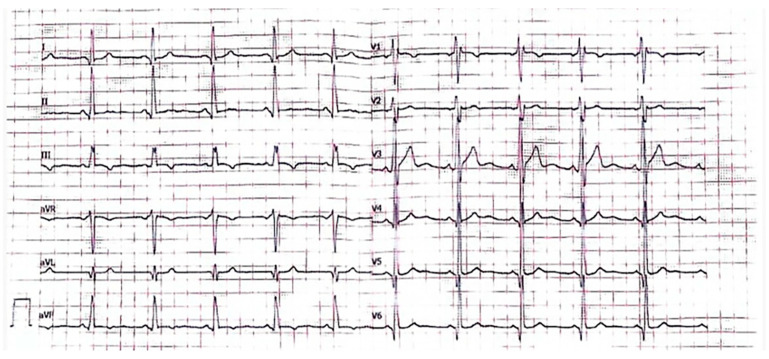
ECG: Sinus rhythm, short PR interval (110 ms), incomplete right bundle branch block.

**Figure 2:**
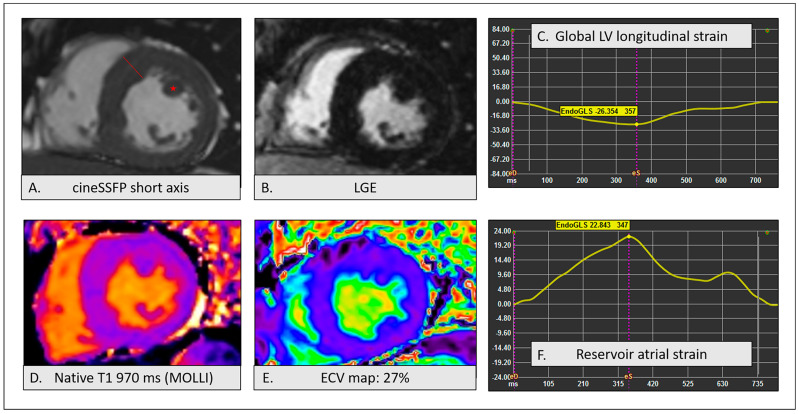
Cardiac magnetic resonance findings: (**A**) Cine steady-state free precession (SSFP) short-axis image showing maximal wall thickness (17 mm—red line) of the anterior interventricular septum and anterolateral papillary muscle hypertrophy (red star); (**B**) negative late gadolinium enhancement (LGE) sequence; (**C**) cardiac magnetic resonance (CMR) feature-tracking left ventricular (LV) strain: normal LV global longitudinal strain (−26.3%); (**D**) modified Lock-Locker inversion recovery (acronym MOLLI) sequence for T1 mapping revealing normal native T1 at the interventricular septum (970 ms); (**E**) extracellular volume (ECV) map showing normal values of extracellular volume (27%) at the interventricular septum; (**F**) CMR feature-tracking LV strain: normal atrial reservoir strain (22.8%).

We describe the case of a 44-year-old Caucasian male of Bulgarian origin, former smoker, with early-onset arterial hypertension (AH) up to 180/90 mmHg, undergoing routine cardiovascular assessment for recurrence of syncopal episodes. The ECG showed sinus rhythm, short PR interval and incomplete right bundle branch block (RBBB) (Figure 1). Echocardiography revealed concentric LVH with a maximal wall thickness (MWT) of 17 mm at the interventricular septum, with normal biventricular function. The patient was therefore referred to cardiac magnetic resonance (CMR) to further evaluate the tissue characterization and to validate the finding of LVH. CMR showed normal LV volumes, supra-normal LV ejection fraction (EF) without alterations in segmental kinetics and LVH with increased LV mass. A MWT of 17 mm was confirmed at the mid-interventricular septum and hypertrophy of the anterolateral papillary muscle was also evident. In cine steady state free precession (SSFP) images, a subaortic signal demodulation suggested dynamic flow acceleration at the LV outflow tract. Tissue characterization sequences, including T2w, late gadolinium enhancement (LGE) and multiparametric mapping images, provided no evidence of tissue alterations such as either myocardial edema or fibrosis. LV longitudinal strain and atrial reservoir strain were within normal limits, excluding subclinical systolic and diastolic dysfunction, respectively. CMR findings are depicted in Figure 2.

Given the effective pharmacological control of the blood pressure and atypical electrocardiographic (ECG) alterations for LVH, the patient was referred by the cardiovascular imaging expert for assessment by a geneticist. After genetic counseling, no couple consanguinity emerged among the four grandparents nor between the patient’s parents. His mother was treated in Turkey for ischemic and hypertensive cardiomyopathy, and she died at the age of 56 after several syncopal episodes; no autopsy data are available. A maternal aunt experienced sudden cardiac death (SCD) at the age of about 50 and unfortunately, also in her case, autopsy was not performed. His sister was affected by early-onset AH and diagnosed with hypertensive heart disease in Bulgary. The patient’s offspring includes (i) a 9-year-old son in good health, practising agonistic sport activity (football), with normal ECG (Supplementary File Figure S1) and echocardiography for age, and (ii) a 14-year-old daughter with lipothymic episodes, ECG alterations (short PR interval, negative T waves in v3–v6 and inferior leads—Supplementary Files Figure S2) and echocardiography within normal limits for age.

### 2.2. Results and Discussion

**Figure 3:**
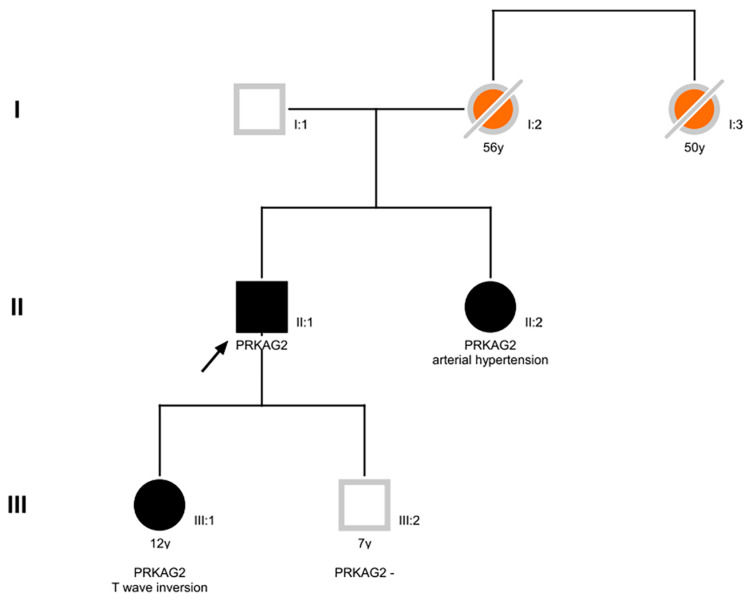
Family pedigree (the family pedigree has been drawn using the Pedigree XP Software Version 2.6.0.209 lisenced to IRCCS Policlinico San Donato until 1 September 2024): The proband is indicated by the black arrow. Circles represent females, and squares represent males. Filled-in figures indicate affected members. A slash through the symbol represents deceased individuals; I, II and III represent family generations.

Genetic testing on the proband peripheral blood explored a panel of 46 genes associated with HCM and revealed the missense variant c.1006G>A p. (Val336Ile) in a single copy of the *PRKAG2* gene, with unknown inheritance. The same variant was identified in the patient’s sister (in Bulgary) and his daughter. The family pedigree is depicted in Figure 3. This missense variant, absent in general population databases, is located next to the AMP-binding CBS1 domain, replacing the highly conserved valine 336. This variant has been previously reported in some individuals (ClinVar database, ID 179205) without any phenotype correlation. In silico prediction, software does not agree on the possible impact of the amino acidic substitution: indeed, Revel, SIFT and FATHMM are inconclusive, whereas MetaLR, Mutation Taster and DANN predict a damaging effect. Overall, the aggregate prediction of the p. (Val336Ile) change computed by Franklin software (Version 12 January 2022 licensed by GENOOX company based on free resources such as dbVar, UMLS, HPO, Orphanet—https://franklin.genoox.com/clinical-db/home (accessed on 10 July 2024)) impact is reported as deleterious. Conversely, SpliceAl (Version 20 June 2024 based on hg38 build), software based on deep neural networks, did not predict a splicing effect of the c.1006G>A substitution.

A very similar variant affecting the same amino acid (p. Val336Leu) was previously reported by Kun-Qi Yang et al. [6], in a Chinese family, where the affected members showed diffuse concentric hypertrophy, one case of apical hypertrophy, markedly increased LV mass and prominent LGE in 3/5 patients, with a predominant subendocardial pattern in mid-distal segments. CMR findings were associated with various combinations of ventricular pre-excitation, conduction disturbances and AH. The authors described a three-generation pedigree and found the heterozygous single-base alteration in five members and only one young asymptomatic carrier.

Another *PRKAG2* mutation affecting the same amino acid (p. Val336Ala) was identified in a Caucasian family [7], displaying a severe cardiac phenotype, characterized by paroxysmal atrial fibrillation (AF), atrioventricular block and massive LVH, associated with SCD. In addition, other causative mutations were identified in the region between CBS1 and CBS2 [8] in Caucasian patients with LVH, [c.1004T>C (p. Met335Tyr); c.1023A>T (p. Leu341Phe)] [9], hinting at the critical function of this site, likely to be a mutational hotspot.

Therefore, several pieces of evidence support a likely pathogenic role of this variant: it is absent in population databases (gnomAD, ExAc), it is located adjacent to a functional domain (AMP-binding CBS1 domain), it involves a highly conserved amino acid from human to Zebrafish (UCSC genome browser), it is predicted damaging by different in silico software, different substitutions of the Val336 were reported in other HCM patients and the variant segregates with the disorder in our family.

In this report, we describe the genotype–phenotype correlation between the likely pathogenic heterozygous variant c.1006G>A p. (Val336Ile) in *PRKAG2* gene and Caucasian patients’ clinical pictures. This is characterized by non-massive LVH, absence of myocardial fibrosis, early-onset AH and prominent conduction disturbances.

At a first glance, these findings could easily be attributed to hypertensive cardiomyopathy, but, notably, AH is a very well-known feature of *PRKAG2* clinical spectrum.

Thus, in our case, AH is likely to be considered as associated with the syndrome, especially when AH onset is early and in presence of familiar recurrence. Indeed, AH might play a role in the development and progression of LVH.

The CMR phenotype of our patient differs from the cases reported by Kun-Qi Yang et al. [6] and could be interpreted as a form of late-onset mild LVH [10].

During the first months of follow-up, the patient developed tachy–brady syndrome and, in consideration of the risk profile [11] (familiar history of SCD, short PR interval, RBBB, LVH, supraventricular tachycardia, early-onset AH), he was implanted dual-chamber implantable cardioverted defibrillator (ICD). It is remarkable that, despite a mild hypertrophic phenotype, with limited evidence of structural involvement, preserved ventricular function and non-massive LVH, the arrhythmic phenotype of the proband was predominant. This is in contrast with what has been previously published among patients harboring *PRKAG2* heterozygous variants [11]. Given the family history, including a case of sudden cardiac death and several instances of syncopal episodes across family members, this new genotype–phenotype correlation is noteworthy.

In conclusion

(1) The proband imaging highly resembled a hypertensive cardiomyopathy, but a multidisciplinary discussion prompted further evaluation.
(2) The discrepancy between the extent of AH and the limited structural involvement contrasting with both family history and ECG phenotype (short PR and incomplete right bundle branch block) addressed genetic testing.
(3) A prevalent arrhythmic and conduction disturbance-related phenotype is associated with c.1006G>A *PRKAG2* heterozygous variant in this family, providing a new genotype–phenotype correlation.

**Figure 4:**
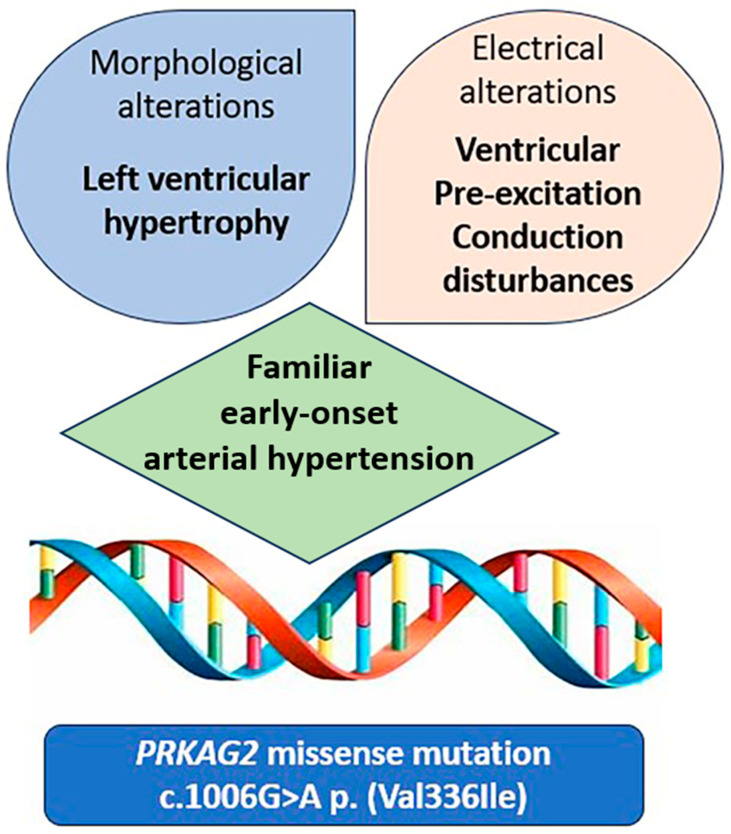
Central illustration: The coexistence of left ventricular hypertrophy, ECG alterations and familiar early-onset arterial hypertension directed genetic testing that identified the missense mutation c.1006G>A p. (Val336Ile) in *PRKAG2* gene.

Paying close attention to the coexistence of LVH, ECG alterations and familiar early-onset arterial hypertension (Figure 4) helped direct diagnostic investigations to exclude primary cardiomyopathy. Identifying the genetic basis in the proband allowed for familial screening as well as a proper follow-up and therapeutic management of the patient. Serial CMR imaging during follow-up could be useful for evaluating the progression of LVH and the development of tissue alterations associated with this mutation in all affected members. Currently, *PRKAG2* cardiomyopathy has shown extremely heterogeneous phenotypes, the progression of which over time is still poorly understood, especially regarding phenotype–genotype correlations. We enclose below a literature review, showing the state of the art regarding *PRKAG2* syndrome, to complete our dissertation about the topic.

## 3. PRKAG2 Syndrome Literature Review

### 3.1. History and Genetics

The first description of the association between LVH and ventricular pre-excitation dates to 1966 [12]. Later on, in 1991, PRS was mapped in the locus 7q 36 [13]. Still, only in the early 2000s, the development of gene sequencing technologies discovered that mutations in genes involved in glycogen metabolism were responsible for the syndrome [14]. The first report of *PRKAG2* causative mutation occurred in 2001 [5], and to date, nine pathogenic mutations are reported in Leiden Open Variation Database (LOVD, last updated in 19 April 2024, genomic reference NG_007486.1). However, there is plenty of variants that do not fulfill the criteria for pathogenicity and are thus classified as variants of unknown significance (VUS); in the study from Lopez-Sainz et al. [10], VUS accounted for 30% of cases.

A systematic review, including 193 *PRKAG2* patients from 23 published studies [11], reported c.905G>A (Arg302Gln) and c.1463A>T (Asn488Ile) as the most common mutations, with 110 and 40 cases, respectively. Patients harboring c.905G>A show a greater prevalence of ventricular pre-excitation, syncope and early pacemaker (PM) implantation, while LVH is more frequently observed among patients with c.1463A>T mutation.

Five *PRKAG2* heterozygous mutations [15] (p. Arg531Gln, p. Glu506Gln, p. Arg384Thr, p. Lys475Glu and p. Gly100Ser) were associated with early onset of HCM and poor prognosis. Among those, the most severe is c.1592G>A (Arg531Gln), with a report of cardiogenic shock and death within the first three months of life [16]. Also two novel variants, Leu341Ser and Lys485Glu, have been associated with severe cardiac phenotype requiring heart transplantation at a young age [17]. The p.Glu506Lys mutation was described in a Turkish family with PRS and associated with progressive HCM leading to heart failure (HF) with reduced EF at 40 years of age [18]. p.Ser333Pro, p.Val336Ala and p.His530Arg mutations were reported in a French cohort of 34 PRS patients, the latter being associated with early evolution towards HF [7].

Systemic involvement with skeletal myopathy and creatine phosphokinase (CPK) elevation was observed among patients with c.1463A>T (Asn488Ile) or Ser548Pro [19], while the most frequent mutation (Arg302Gln) was never associated with extracardiac involvement. Early presentation with LVH and skeletal muscle involvement were also reported in the presence of c.1518A>C (p. Glu506Asp) or c.1463A>T (p. Asn488lle) mutations [20].

Association with early-onset AH occurred with c.1591C>G (p. Arg531Gly) and c.1453A>G mutations [21].

In patients with non-sarcomeric LVH and Wolff Parkinson White (WPW), almost all studies document missense mutations, except for Blair et al. [22] who reported an insertion mutation (Exon 5:InsLeu) associated with LV systolic dysfunction. Biallelic *PRKAG2* truncating variants were associated with severe and fatal neonatal cardiomyopathies [23]. The missense mutation c.425C>T (p.T142I) heterozygous variant was associated with a dilated cardiomyopathy (DCM) early-onset phenotype [24]. A case report described a combination of *SCN5A* p.A204E and *PRKAG2* p.D372N mutations associated with DCM, LVH and multifocal ectopic Purkinje-related premature contractions [25].

The association between LVH, conduction abnormalities and LV non-compaction [26,27] was reported in two Chinese families.

Table 1 reviews all previous reports on *PRKAG2* cardiomyopathy, with associations between genotype and phenotype.

### 3.2. Pathophysiology

*PRKAG2* gene encodes the γ2 regulatory subunit of 50 Adenosine Monophosphate-Activated Protein Kinase (AMPK), which is a highly conserved serine/threonine protein kinase [67] that modulates cellular energy homeostasis by switching on ATP-generating pathways and turning down anabolic pathways, in response to cellular stress. AMPK is extensively expressed in cardiac tissue, where it regulates glucose and fatty acid uptake, storage and employment. Mutations in *PRKAG2* cause structural changes in AMPK and alter its affinity to AMP, impairing carbohydrate metabolism and ultimately causing storage cardiomyopathy with glycogen deposition within myocites [68]. Most disease-causing mutations are observed in the highly conserved CBS domain region, disrupting the typical interaction between the AMPK-γ2 subunit and adenosine-containing ligands.

Studies conducted in vitro and on animal models yielded conflicting results regarding the activation of AMPK in the presence of *PRKAG2* mutations [16,64,69,70,71,72,73,74]. However, regardless of the specific molecular mechanism, all these mutations led to glycogen accumulation. Unlike fiber disarray and myocardial fibrosis, typical of sarcomeric HCM, the histopathologic feature of *PRKAG2*-related myocardial hypertrophy is collagen-filled myocyte vacuolations [19,21,68]. However, there is evidence suggesting that not only deposits but also disorders in ATP handling [19] may contribute to tissue damage, potentially affecting skeletal muscles. Interesting insights in PRS molecular derangements derive from animal models. In a mouse model of mutant *PRKAG2*, in which glycogen deposition was inhibited, Kim et al. [75] observed that storage ablation eliminated LV pre-excitation but did not affect cardiac hypertrophic growth, which is modulated by enhanced insulin sensitivity and protein kinase B activation. Banerjee et al. [76] showed that dysregulated AMPK activity triggers the early activation of NF-KB and AKT signaling, inducing myocardial hypertrophy. Also, mutated AMPK [77] was found to be related to autophagy and apoptosis [78] and could unbalance the phosphorylation state of cardiac troponin, impacting myocardial contractility.

Isolated histological reports of interstitial fibrosis, myofibrillar disarray and fibrofatty replacement in PRS are present in the literature [21,79].

As for conduction disturbances, the pathological mechanisms are still unclear. Glycogen-filled myocytes may hinder the physiological atrioventricular septation during cardiogenesis [5,60,80], causing pre-excitation and reciprocating arrhythmias. In transgenic mice, glycogen deposition damaged the annulus fibrosus and led to WPW [70]. Inhibition of glycogen content accumulation in cardiomyocytes effectively suppresses arrhythmias in transgenic mice [81]. However, Tan et al. [82] found Mahaim fibers in a PRS patient (R302Q) with SCD, in the absence of glycogen accumulation. Finally, other studies showed that AMPK mutation could lead to ionic channel dysfunction and arrhythmia [83,84].

### 3.3. Clinical Features

In the OMIM website, there is a specific entry of a metabolic disease, causing altered glycogen storage due to heterozygous mutations of the *PRKAG2* gene, with a clinical picture characterized by systemic involvement [64]. PRS phenotype varies between families, and even family members carrying the same mutation may display different clinical manifestations, indicating variable expressivity of the disease [19,20,27,29]. The penetrance reported for PRS is as high as 99% [11]. The onset of symptoms usually occurs between the third and fifth decades of life, albeit intrauterine development of the disease has been reported [16], as well as cases of late-onset mild LVH [10].

- Left ventricular hypertrophy and function:

PRS typically includes progressive symmetric LVH, with variable MWT. Although severe LVH has been classically depicted, ranging from 24 mm in Asn488Ile to 33 mm in Glu506Lys [7], an MWT > 20 mm was only reported in fewer than one half of patients from a multicentric European cohort [10]. LVH occurs more commonly in a concentric pattern [25,47,48,49,50]; however, eccentric LVH with predominant involvement of the interventricular septum has been reported, as well as early syndromes exhibiting infero-lateral wall LVH and evolving towards a diffuse pattern [8]. Cases of asymmetric localized LVH [58,85], apical hypertrophy with “spade-like” cavity and papillary muscle hypertrophy have also been described [17,29]. Concomitant right ventricular (RV) hypertrophy may be present [39,54,86]. Right atrial wall thickening has been detected in the advanced stages of the disease [39].

Demand–supply mismatch myocardial ischemia [58] has been observed in the case of massive LVH.

In most patients, the natural history of *PRKAG2* mutations reveals a slowly progressive increase in wall thickness [19]; while, in sarcomeric HCM, LVH undergoes a gradual decrease with aging. Moreover, patients with sarcomeric HCM usually present with asymmetrical LVH predominantly involving the interventricular septum [87].

*PRKAG2* cardiomyopathy may evolve from LVH with preserved EF towards LV dysfunction and dilation, resembling DCM. Both diastolic and systolic dysfunction may progressively develop [34,86]. HF affects 12% of patients [11], with a significantly higher prevalence in the presence of Glu506Lys (exon5:InsLeu) [22] and His142Arg mutations [18]. In the largest European cohort, systolic dysfunction was observed in more than 20% of patients [10]. In presence of preserved LV EF, reduction in global longitudinal strain (GLS) was reported [54,82]. RV systolic dysfunction by longitudinal strain and 3D EF were also observed [29,86], even in absence of PM [88]. Advanced HF complication rates are worse in patients with PRS than in other patients with HCM [89].

LV outflow tract obstruction (LVOTO) is rare [10,29,38,86], if present, and together with a restrictive diastolic pattern and progression towards LV dilation, they represent a leading cause of adverse outcomes [22] (i.e., SCD and cardiac transplant). Few cases of heart transplants are reported, especially in patients with the most common variants [14,16,60].

A single case report described the association between LVH, WPW and coronary bridging causing angina in a 24-year-old female with *PRKAG2* Arg302Gln mutation and a heterozygous *CACNB2* mutation on exon 4 (VUS) [48].

- Ventricular pre-excitation:

ECG alterations in the absence of ultrastructural or echocardiographic changes have been reported with a 100% penetrance before 18 years of age, as compared to a 78% penetrance of LVH after 18 years of age [19]. The typical ECG feature of PRS is a short PR interval, which is reported in 68% of patients [14] and is indicative of pre-excitation. In a South Asian cohort, a typical WPW pattern with short PR interval and delta wave was observed in 77% of PRS patients [38]; while, in the European cohort by Lopez Sainz et al. [10], the pre-excitation pattern was only detected in one-third of the population. The pre-excitation ECG pattern is most commonly subtended by common accessory pathways and decremental atrioventricular connections or fasciculoventricular pathways [14,19,90]. Different accessory pathways responsible for reciprocating atrio-ventricular tachycardia have been described in Arg302Gln patients [22,91,92]. Patients with accessory pathways associated with *PRKAG2* mutations often have distinct clinical, ECG and electrophysiologic profiles [45,60], and radiofrequency ablation may lead to iatrogenic atrio-ventricular block [19].

- Conduction system abnormalities and arrhythmias:

Overall conduction system alterations occur in 44% of PRS patients, and over 40% of patients are implanted with a permanent PM within the third or fourth decade of age [11]. The mean onset age for symptomatic conduction disease is 38 years, ranging from 16 to 56 years [11,19]. However, conduction disturbances are not always present in PRS and may occasionally develop later in life [91,92]. Typical ECG features of PRS are RBBB, slurred QRS depolarization phases and eccentric patterns of intraventricular delays >120 ms. Sinus bradycardia, sinoatrial and advanced atrioventricular blocks may cause chronotropic incompetence, correlated to syncope, Adam Stokes syndrome and hemodynamic decompensation, requiring premature PM implantation. Other reported ECG features are high QRS voltage, depolarization abnormalities and left axis deviation, even in the absence of LVH.

Supraventricular arrhythmias (SVT) are reported in 38% of patients with PRS [11]. AF might be the symptom of onset, associated with stroke and rapid ventricular arrhythmias in the presence of pre-excitation. Of note, AF is common and occurs about 10 years earlier than in sarcomeric HCM, with an average age of 43 ± 16 years [10]. There is evidence that the p.Arg302Gln mutation directly damages the atrium, causing intense vacuolization of cardiomyocytes and fibrosis, when comparing samples from mutant left atrial appendage and age-matched individuals with AF [35]. Findings in R302Q PRS patients suggest that atrial glycogen deposition may determine atrial conduction disturbances [47], and atrial enlargement might even precede LVH at earliest stages of the disease [27,93]. In nearly half of patients with early-onset cryptogenic stroke, pathogenic variants in cardiogenic disease genes, including *PRKAG2*, have been identified, indicating cardioembolic susceptibility even in the absence of structural heart abnormalities [94].

The prevalence of SCD in PRS is commonly reported as around 8–10%, with a mean age of 33 years [10,11,19,60,65]. Higher rates have been reported in a South Asia cohort (27%) [65] and in a French cohort [7] (20%). SVT degenerating into ventricular fibrillation (VF) and advanced atrio-ventricular block are among the main causes of SCD [10,60]. Interestingly, electropshysiological study (EPS) in PRS patients showed VF induction by high atrial pacing and not by ventricular extra stimuli [19]. SCD has been reported in PRS patients also in the absence of massive LVH [60] and occasionally during sleep [92].

- Arterial hypertension and extracardiac involvement:

Up to 50% of *PRKAG2* pathogenic mutation carriers are reported to have AH, often with young age onset [17,29]. The association between AH and PRS is not fully understood; however, it is plausible that early-onset AH may accelerate and contribute to LVH.

PRS may occasionally manifest with systemic involvement such as skeletal myopathy and CPK elevation [5,19,21,60]. The observed discrepancy between the severity of cardiac and skeletal muscle symptoms may be due to a greater expression of the AMPK-γ2 subunit in the heart [14]. In some cases, ventricular pre-excitation and skeletal myopathy precede the development of LVH.

### 3.4. Cardiac Magnetic Resonance Findings

CMR is the gold standard for evaluating ventricular function, volumes and wall thickness, in addition to providing non-invasive tissue characterization. Hence, CMR is the ideal technique for studying metabolic and infiltrative cardiomyopathies. To date, only few cases of *PRKAG2* cardiomyopathy have been described by CMR, and no specific pattern has been identified [6,8,54,58,85]. It is plausible that different mutations in the *PRKAG2* gene may result in phenotypic heterogeneity, similar to what occurs in sarcomeric HCM. CMR documented that LVH is commonly symmetric, although cases of asymmetry with predominantly septal, mid-inferolateral or distal involvement have been described, as well as rare cases of RV involvement [39,54,86]. Myocardial hypertrophy of the infero-lateral wall has been described in the early phases of the disease, while a more diffuse LVH pattern with prevalence at the interventricular septum has been reported in the advanced stages [8]. However, in some patients, an increase in LV volumes and/or a progressive deterioration of LV EF have also been reported.

Data on tissue characterization are heterogeneous [6,8,38]. In a South Asian cohort with p.Arg302Gln by Ahamed et al. [38], LV LGE was observed in a minority of patients, while Yang et al. [6] documented a greater prevalence of LGE, including one case of RV LGE. It is plausible that different mutations may entail different fibrotic burdens and/or patterns. Also, findings may change according to the stage of progression of the disease, since fibrotic burden seems to progressively increase with the degree of LVH [29,38]. Subendocardial and patchy or ill-defined intramyocardial LGE distributions are the most commonly described, and some cases or transmural LGE have also been reported [6,39,41,54]. Heterogeneity in LVH and LGE patterns may be present also within the same family [39]. Yang et al. [6] reported an association between LGE and adverse clinical outcomes such as SCD, worsening of LV EF, ventricular dilation and conduction disturbances.

In patients without LGE, T1 values may be reduced at the very early stages and progressively increase with the development of myocardial fibrosis [8]. The presence of interstitial fibrosis has also been reported as an increase in extracellular volume fraction (ECV) [39].

A CMR study [95] comparing HCM with rare phenocopies, including PRS, showed that LGE extension, global native T1 and ECV are significantly higher in patients with rare diseases.

### 3.5. Differential Diagnosis

Differential diagnosis between hypertrophic phenocopies is of paramount importance, as it allows the acquaintance of the specific natural history of the disease, as well as addressing clinical management and medical treatment. Early diagnosis may allow extra support for daily activities and assistance to alleviate the psychological impact of the disease.

Differential diagnosis between *PRKAG2* cardiomyopathy and HCM is based on genetic analysis, as the two diseases may overlap, although the phenotypic expression of both phenocopies is highly variable. Other differential diagnosis for PRS are Danon’s cardiomyopathy and Anderson–Fabry cardiomyopathy, which are both X-linked genetic disorders. Danon’s cardiomyopathy [36] is characterized by massive LVH, short PR interval, high arrhythmic burden and a mean survival rate <25 years; extracardiac involvement consists of skeletal myopathy and intellectual disability. Cardiac involvement in Anderson–Fabry disease [96,97,98] includes reduced native T1 due to intracellular lipid storage, LVH and myocardial inflammation/fibrosis of the infero-lateral basal wall; other extracardiac symptoms are acroparesthesias, renal failure, stroke, abdominal pain, angiokeratomas and corneal-lenticular opacities. Also, mitochondrial disorders [99] may be associated with HCM phenocopies.

The prognosis of patients with *PRKAG2* genetic variants is better than that of patients with Danon disease, especially for men, although it is still poorer compared to those with sarcomeric HCM [89,100].

Differential diagnosis with Danon or Fabry cardiomyopathy is pivotal since enzyme replacement therapy (ERT) may be implemented in these latter cases.

### 3.6. Clinical Management and Treatment

Since no specific guidelines for *PRKAG2* cardiomyopathy are available, the approach to the disease commonly refers to the ESC guidelines for the diagnosis and management of HCM [101].

As for HCM, MWT and LV EF at baseline proved to be prognostic markers. Of note, in the largest European cohort reported in the literature [10], the mean LV EF of patients with events was 55%, indicating that borderline values may already entail clinical implications. Porto et al. suggested a red flags-based approach [11] including familiar history (SCD, genetic disease with AD inheritance), age (young onset between the first and the fourth decades), ECG (signs of pre-excitation, bradycardia, atrioventricular blocks, bundle branch blocks), echocardiography (LVH), ECG monitoring (SVT, chronotropic incompetence), EPS (presence of accessory pathways) and systemic involvement (myalgia, early-onset AH, epilepsy). Other potential prognosticators that may aid risk stratification are syncope with arrhythmic features, extent of LVH, LGE and documented non-sustained ventricular tachycardia (NSVT).

In suspicion of PRS, genetic counseling and accurate familiar screening are recommended. The mutation in the proband can be leveraged for family screening to identify gene carriers for strict follow-up and preventive measures. Moreover, the identification of *PRKAG2* pathogenic variants also allows preimplantation or prenatal genetic testing.

Genetic testing in *PRKAG2* is not a strategy to predict prognosis, since no differences between variants were identified in terms of adverse disease-related events [10]. Strict arrhythmic monitoring is advised, especially in young patients with clinical suspicion of brady- or tachyarrhythmias. Exercise stress testing and EPS may also be useful to investigate the mechanism and the clinical context of arrhythmias and possibly consider PM implantation in the presence of advanced atrioventricular block or chronotropic incompetence.

Arrhythmic risk stratification and indications of ICD implantation in primary prevention are still debated, due to the lack of follow-up data and the small number of events reported in the literature; moreover, SCD RISK score is not applicable to HCM phenocopies. The decision should be weighted by taking into consideration red flags such as familiar history, documented NSVT/VT, syncope of suspected arrhythmic origin and high-risk patterns of pre-excitation at EPS. A dual-chamber ICD should be evaluated in PRS patients in presence of conduction system disease.

Lastly, CMR may contribute to patient stratification through tissue characterization and accurate quantification of LVH and LGE extent.

Although there is no specific treatment for PRS yet, it might represent a suitable candidate for ERT or gene therapy, as already happened for other storage HCM phenocopies. Promising pre-clinical studies show that glycogen-storage cardiomyopathy and conduction system degeneration associated with *PRKAG2* mutation might be reversible, and the development of accessory pathways may be prevented by inhibition of glycogen accumulation during early postnatal development [81].

The prognosis of HCM phenocopies associated with glycogen metabolism defects is usually worse than that of sarcomeric protein gene variants [7,11]. Early diagnosis of PRS and application of contemporary therapies may hopefully bring *PRKAG2* cardiomyopathy towards the low mortality rates and good quality of life that have been reached over the years for sarcomeric HCM.

## 4. Materials and Methods

Genomic DNA was extracted using a Maxwell automatic extractor (Promega Corporation, 5500 East Cheryl Parkway Suite 110, Feynman Center Manufacturing 2780 Woods Hollow Rd, Madison, WI, USA), enriched with TruSight One Expanded (Illumina, 5200 Illumina Way, S. Diego, CA 92122, USA) and sequenced by Next-Generation Sequencing (NGS) on NextSeq2000 platform (Illumina). Sequences were analyzed according to GATK Best Practice criteria, exploiting pipelines based on BWA, Smith–Waterman algorithm, Free Bayes, SnpSift–SnpEFF and Base Space Onsite. Variants in a panel of 46 genes (*ANKRD1*, *ACTC1*, *ACTN2* (*except intervals g.236897733-236897818 and g.236899858-236899968*), *BAG3*, *BRAF*, *CACNA1C*, *CAV3*, *COX15*, *CSRP3*, *DES*, *FHL1*, *FHOD3*, *FLNC*, *GAA*, *GLA*, *HRAS*, *JPH2* (*except interval g.42805457-42806612*), *KLF10*, *KRAS*, *LAMP2*, *LDB3*, *MAP2K1*, *MAP2K2*, *MYBPC3*, *MYH6*, *MYLK2*, *MYOM1*, *MYOZ2*, *MYPN*, *MYH7*, *MYL2*, *MYL3*, *NRAS*, *PLN*, *PRKAG2*, *PTPN11*, *RAF1*, *RIT1*, *SHOC2*, *SLC25A4*, *SOS1*, *TNNC1*, *TNNI3*, *TNNT2*, *TPM1* and *TTR*) associated with cardiomyopathies were prioritized based on information in public databases (ex. gnomAD, NCBI, LOVD) and classified according to ACMG guidelines [9]. Candidate variants were confirmed by Sanger sequencing. The presence of the candidate variants in family members was evaluated by Sanger sequencing.

## Figures and Tables

**Table 1:** Review of previous reports on *PRKAG2* cardiomyopathy with genotype–phenotype correlation.

Study	Nr Patients	Genetics	Imaging	Other Findings
Oktay et al., Anatol J Cardiol 2023 [28]	2	c.634C>T VUS novel	Echo or CMR: LVH.	-
Janin et al., Circ. Genom Precis Med. 2023 [23]	3	2 pts: homozygous truncating variant: p.Ile550Asnfs*58 1 pt: homozygous 2504 base pair deletion removing exon 11	Echo: dilated hypokinetic cardiomyopathy. Autopsy: dilated ventricles, thin LV wall with fibrosis and fibroelastosis.	Neonatal cardiogenic shock. ECG at 4 months of age: positive T waves in V1 and V2, negative T waves in V3 to V6, fragmented P wave, slightly prolonged PR interval.
Marcu et al., Life 2022 [29]	4: same family	p.Arg302Gln heterozygous	Echo and CMR: 1 pt concentric biventricular hypertrophy, hypertrophied anterolateral papillary muscle, apical hypertrophy. No LGE, normal T1; 1 pt no LVH; 1 pt LVH only; and 1 pt not available.	Early-onset AH, short PR, WPW, AF, PM 4th decade for advanced AV block, heart failure.
Cadena-Ullauri et al., Front Cardiovasc Med, 2022 [30]	2: same family	c.905G>A p.Arg30Gln	1 pt Echo: structurally normal heart; and 1 pt not available.	ECG signs of LVH, WPW, migraines and tachycardia.
Pena et al., Arq Bras Cardiol, 2022 [31]	30	28 pts Arg302Gln 2 pts His401Gln	Echo: reduced RVEF in 56.7% of pts, RVEF below 35% in 7 pts.	WPW, AH, atrial flutter and AF.
Zhang et al., BMC Med Genomics, 2022 [26]	5: same family	c.905G>A (p.R302Q)	1 pt CMR: LVH and LVNC; 2 pts CMR: LVNC; 2 pts Echo: normal.	Short PR, WPW, sinus bradycardia, 2 pts with normal ECG.
Magalhães et al., Arq Bras Cardiol, 2022 [32]	5: same family	Arg302Gln	Echo: LVH in 4 pts.	Atrial flutter as clinical onset, WPW, RBBB, PM 4th decade for advanced AV block.
Komurcu-Bayrak et al., Archives of Biochemistry and Biophysics, 2022 [33]	8: same family	E506K	Echo: LVH in all.	WPW, PM, SCD in 2 pts.
Huang et al., Front Cardiovasc Med, 2022 [25]	2 siblings	p.D372N (+*SCN5A* p.A204E)	1 pt Echo: dilated and hypokinetic LV; and 1 pt Echo: dilated and hypokinetic LV, LVH.	1 pt SCD and 1 pt HTx (10 yo).
Gong et al., Mol Genet Genomic Med, 2022 [24]	2	1 pt c.425C>T (p.T142I) 1 pt c.869A>T (p.K290I)	1 pt Echo and CMR: dilated and hypokinetic LV, no LGE; and 1 pt Echo and CMR: asymmetric LVH.	1 pt HF (9 yo) and 1 pt WPW, syncope (10 yo).
Tang et al., Cardiovascular Ultrasound, 2022 [34]	9	-	Echo: LVH.	Intellectual disability, WPW, reduced GLS.
Chen et al., Front Cardiovasc Med, 2022 [35]	3	c.905G>A (R302Q)	2 pts Echo: LVH; 1 pt echo normal.	Atrial flutter, AV block, short PR, PM, thrombus in the LAA, syncope.
Fang et al., J Clin Med, 2021 [36]	1	c.166G>A (p.G56S) c.298G>A (p.G100S)	CMR: HCM, extensive mid-myocardial LGE > lateral wall.	Syncope, ICD, heart failure, death.
Beyzaei et al., BMC Med Genomics, 2021 [37]	1	c.592A > T (p.Met198Leu)	-	Liver cirrhosis.
Ahamed et al., Sci Rep, 2020 [38]	22	c.905G > A (p.Arg302Gln)	Echo: 19 pts biventricular hypertrophy. CMR: 2/8 pts with LGE.	WPW, PM for AV block and/or sino-nodal disturbances, AF, 6 pts SCD or equivalent.
Hu et al., Curr Med Sci, 2020 [27]	5	Arg302Gln	Echo: 3 pts asymmetric LVH and 1 pt symmetric LVH, atrial enlargement. CMR in 2 pts: no LGE, 1 pt apical LVNC.	WPW, PM for AV block and severe bradycardia.
Lopez-Sainz et al., JACC, 2020 [10]	90	32 pts p.Arg302Gln 30 pts p.Asn488Ile + rare variants.	Echo: 60 pts LVH.	WPW, PM, AF, heart failure, 3 pts SCD, 4 pts aborted SCD, 11 pts death/HTx.
Wang et al., Int J Cardiovasc Imaging, 2020 [39]	2 siblings	c.866A>G (p.His289Arg)	1 pt Echo and CMR: biventricular hypertrophy, massive LVH, increased native T1 and ECV, patchy LGE, reduced GLS; and 1 pt Echo and CMR: LVH, increased native T1, subendocardial to mid wall LGE at the basal inferior wall.	WPW.
Hu et al., EBioMedicine, 2020 [17]	25	R302Q (c.905G>A) R302P (c.905G>C) L341S (c.1022C>T) H401D (c.1201C>G) K485E (c.1453A>G)	Echo: LVH.	WPW, AH, syncope, ICD, PM, AF, PSVT, 1 pt HTx, 1 pt septal myectomy, 2 pts SCD.
Coban-Akdemir et al., Am J Med Genetic A, 2020 [40]	1	c.359G>A, p.Arg120His	-	WPW, Ebstein anomaly.
Hoss et al., Circ Genom Precis Med, 2020 [41]	2	p.Arg302Gln	Echo: asymmetric LVH; and 1 pt CMR: severe LGE+.	WPW and PM for AV block.
Di Stolfo et al., Mol Genet Genomic Med, 2019 [42]	1	Deletion of chromosome 7q35-36 including loss of one copy of *SHH, KCNH2* and *PRKAG2*	Echo: normal.	Multisystemic involvement, dysmorphisms and systemic malformations. ECG: short PR, long QT, VSD (6 yo).
Sri et al., Case Rep Pediatr, 2019 [20]	7: two families	c.1463A>T (p.Asn488lle) c.1518A>C (p.Glu506Asp)	Echo and CMR: LVH, no LGE. Echo and CMR: LVH and 1 pt with LGE.	WPW, skeletal myopathy.
Hata et al., J Clin Med, 2019 [43]	1	p.G75A (VUS)	-	Died accidentally at 72 yo, autopsy: myocyte disarray, AH.
Jääskeläinen et al., ESC Heart Fail, 2019 [44]	1	c.905G>A p.Arg302Gln	Echo: LVH.	-
Gorla et al., J Pediatr Genet, 2018 [15]	1	c.1592G >A (p.Arg531Gln)	Echo: biventricular hypertrophy.	Severe neonatal HCM, fetal hydrops, short PR. Neonatal SCD.
Epicoco et al., JACC: clinical electrophysiology, 2018 [45]	1	His530Arg	Echo and CMR: symmetric LVH.	WPW, atrial flutter, PM/ICD for AV block.
Zhan et al., J Mol Cell Cardiol, 2018 [46]	2 siblings	c.905G>A (R302Q)	1 pt Echo: LVH; and 1 pt Echo: normal.	1 pt PM for AV block, 1 pt WPW.
Sternick et al., Europace, 2018 [47]	1	R302Q	-	PM for AV block.
Banankhah et al., BMC Med Genet, 2018 [48]	1	Arg302Gln	CMR: apical and septal LVH.	WPW, NSTEMI for coronary bridging of LAD. LAD unroofing and myectomy.
Mendes de Almeida et al., PLoS One, 2017 [49]	1	c.1234-317T>G	Echo: LVH.	-
Xu et al., Am J Physiol Heart Circ Physiol, 2017 [50]	1	c.1423 A>G (K475E)	Echo: LVH.	Neonatal onset of HCM, short PR.
Yang et al., Sci Rep, 2017 [6]	5	c.1006 G > T (p.Val336Leu)	Echo and CMR: concentric LVH, RVH, 3 pts LGE +.	WPW, AH.
Zhao et al., Int J Mol Med, 2017 [51]	1	p.Gly100Ser (+associated mutation in *MYBPC3*)	Echo: LVH.	-
Thevenon et al., Europace, 2017 [7]	34	p.Arg302Gln p.Glu506Lys p.Val336Ala p.Ser333Pro p.His530Arg	Echo: 15 pts LVH.	WPW, PM, ICD, 2 pts HTx, 11 pts SCD, skeletal muscle symptoms.
Sternick et al., Circ Arrhythm Electrophysiol, 2016 [52]	1	c.905G>A p.Arg302Gln	-	ECG signs of LVH, PM (20 yo), AF.
Austin et al., Mol Genet Metab, 2017 [53]	1	c.298G>A (Gly100Ser/ G100S)	Echo: mild LVH.	Intention tremor, ataxia and mild hypotonia at neonatal age.
Yogasundaram et al., Circ Heart Fail, 2016 [54]	1	p.Arg302Gln (R302Q)	Echo and CMR: biventricular hypertrophy and outflow tract obstruction, apical LGE+.	Syncope, high-grade AV block.
Zhao et al., Int J Mol Med, 2016 [55]	2	c.298G>A p.Gly100Ser (1 pt + associated mutation *MYH7*)	Echo: LVH.	-
Müllertz et al., Europace, 2016 [56]	3: same family	R302Q	-	ECG signs of LVH, WPW, AF, PM for AV block.
Pöyhönen P et al., J Cardiovasc Magn Reson, 2015 [8]	7: two families	c.905G>A (p.R302Q) c.1031A>C (p.H344P)	Echo and CMR: LVH, 2 pts LGE+.	WPW, PM, 1 pt HTx.
Aggarwal et al., Ann Pediatr Cardiol, 2015 [57]	1	c.1589 A>G (p.His530Arg.)	Echo: concentric LVH.	WPW, high-grade AV block, syncope.
Sternick et al., Eur Heart J, 2014 [58]	1	R302Q	Echo and CMR: massive LVH, septal LGE+.	WPW, NSTEMI.
Schofield et al., BMJ Case Rep, 2013 [57]	1	-	Echo: massive LVH.	AF, HTx.
Liu et al., PLoS One, 2013 [21]	1	c.1453A>G (p.Lys485Glu, K485E)	Echo: LVH, reduced LVEF (46%).	Early-onset AH, WPW, AVRT, AF, PM for AV block, worsening HF (awaiting HTx).
Sheffold et al., Clin Res Cardiol, 2011 [59]	3: same family	p.P83S (c.247C>T) (3 pts + associated mutations in *MYBPC3*)	Echo: severe LVH.	-
Sternick et al., Heart Rhythm, 2011 [60]	10	Arg302gln	Echo: 3 pts LVH.	Fasciculo-ventricular pathway, 5 pts complete AV block, AF, atrial flutter, syncope, 1 pt SCD.
Kelly et al., Pediatr Cardiol, 2009 [61]	3	heterozygous E506Q mutation in Exon 14	Echo: LVH, LVOTO, SAM.	Neonatal onset, short PR, 1 pt HTx 29 yo.
Van Belle Y et al., Pacing Clin electrophysiol, 2008 [62]	1	c.1004 T>C	Echo: LVH.	AF, short PR.
Morita et al., NEJM, 2008 [63]	1	His530Arg	Echo: LVH.	-
Akman et al., Pediatr Res, 2007 [64]	1	R384T heterozygous	Echo: severe hypertrophy of LV septal and atrial walls.	Neonatal death.
Sternick et al., J cardiovasc electrophysiol, 2006 [65]	20	Arg302Gln	Echo: 2 pts asymmetric LVH.	Bradycardia, short PR, RBBB, PM, atrial tachyarrhythmias; 3 pts SCD at a young age; and no pts with WPW.
Bayrak et al., Eur J Heart Failure Case report [18]	8	G1606>A	Echo: biventricular hypertrophy, LVOTO.	Short PR, advanced NYHA class, PM, AV block.
Laforet, Neuromuscolar disorders, 2006 [66]		Ser548Pro	Echo: LVH.	Short PR, skeletal muscle glycogenosis.
Burwinkel et al., Am J Hum Gen, 2005 [16]	3	heterozygous R531Q missense mutation	Echo: congenital LVH, biventricular hypertrophy.	Bradycardia, short PR, WPW, recurrent apnea, progressive HF, VF, all pts neonatal death.
Murphy et al., JACC, 2005 [19]	45	Asn488Ile H304 Arg302Gln H363 Arg302Gln H9	Echo: progressive LVH.	Myalgia, proximal myopathy, skeletal muscle biopsy: excess mitochondria and ragged red fibers with minimal glycogen accumulation, PM, AF, SVT, NSVT, 3 pts stroke, 1 pt SCD.
Arad et al., NEJM, 2005 [4]	3	Missense Y487H	Echo: LVH.	Short PR, SCD.
Blair et al., Human Molecular Genetics, 2001 [22]	8: two families	His142Arg missense, insertional mutation TTA after the codon for arginine 109	Echo: LVH.	Short PR or WPW, PM, AF, 1 pt HTx, 3 pts SCD.

In the case of frameshift mutation the symbol * indicates the premature termination of the encoded protein. AF: atrial fibrillation; AH: arterial hypertension; AV: atrio-ventricular; AVRT: atrio-ventricular re-entrant tachycardia; CMR: cardiac magnetic resonance; ECG: electrocardiogram; Echo: echocardiography; ECV: extracellular volume fraction; GLS: global longitudinal strain; HCM: hypertrophic cardiomyopathy; HF: heart failure; HTx: heart transplant; ICD: implantable cardioverter defibrillator; KCNH2: Potassium Voltage-Gated Channel Subfamily H Member 2; LAA: left atrial appendage; LAD: left anterior descending artery; LGE: late gadolinium enhancement; LVEF: left ventricular ejection fraction; LVH: left ventricular hypertrophy; LVNC: left ventricular non-compaction; LVOTO: left ventricular outflow tract obstruction; LV: left ventricular; MYBPC3: cardiac myosin-binding protein C; MYH7: Myosin Heavy Chain 7; NYHA: New York Heart Association class; NSTEMI: no ST elevation myocardial infarction; NSVT: non-sustained ventricular tachycardia; PM: pacemaker; PRKAG2: Protein Kinase AMP-Activated Non-Catalytic Subunit Gamma 2; PSVT: paroxysmal supraventricular tachycardia; pt: patient; RBBB: right bundle branch block; RVEF: right ventricular ejection fraction; RVH: right ventricular hypertrophy; SAM: systolic anterior motion of anterior mitral valve leaflet; SCD: sudden cardiac death; SCN5A: Sodium Voltage-Gated Channel Alpha Subunit 5; SHH: Sonic Hedgehog; SVT: supraventricular tachycardia; VF: ventricular fibrillation; VSD: ventricular septal defect; VUS: variant of unknown significance; and WPW: Wolff Parkinson White.

## Data Availability

The original contributions presented in the study are included in the article/Supplementary Materials, and further inquiries can be directed to the corresponding author/s.

## Supplementary Materials

The following supporting information can be downloaded at https://www.mdpi.com/article/10.3390/ijms25179171/s1.
