# Supplementary material for: When Paying Attention Pays Back: Missense Mutation c.1006G>A p. (Val336Ile) in PRKAG2 Gene Causing Left Ventricular Hypertrophy and Conduction Abnormalities in a Caucasian Patient: Case Report and Literature Review
Source: Int J Mol Sci. 2024 Aug 23;25(17):9171. doi: 10.3390/ijms25179171 (PMC11395525; doi:10.3390/ijms25179171)
Supplement: Supplementary file 1 [file ijms-25-09171-s001.zip › Supplementary Files.pdf]

Supplementary Files

Figure S1: ECG of the proband’s daughter

Sinus rhythm, short PR (95 msec) and negative T waves in v3-v6 and inferior leads.

Figure S2: ECG of the proband’s son (9 years old)

Normal findings for age: sinus rhythm, normal PR interval (110 msec), no alterations of repolarization.
