# Supplementary figures and images for: When Paying Attention Pays Back: Missense Mutation c.1006G>A p. (Val336Ile) in PRKAG2 Gene Causing Left Ventricular Hypertrophy and Conduction Abnormalities in a Caucasian Patient: Case Report and Literature Review
Source: Int J Mol Sci. 2024 Aug 23;25(17):9171. doi: 10.3390/ijms25179171 (PMC11395525; doi:10.3390/ijms25179171)

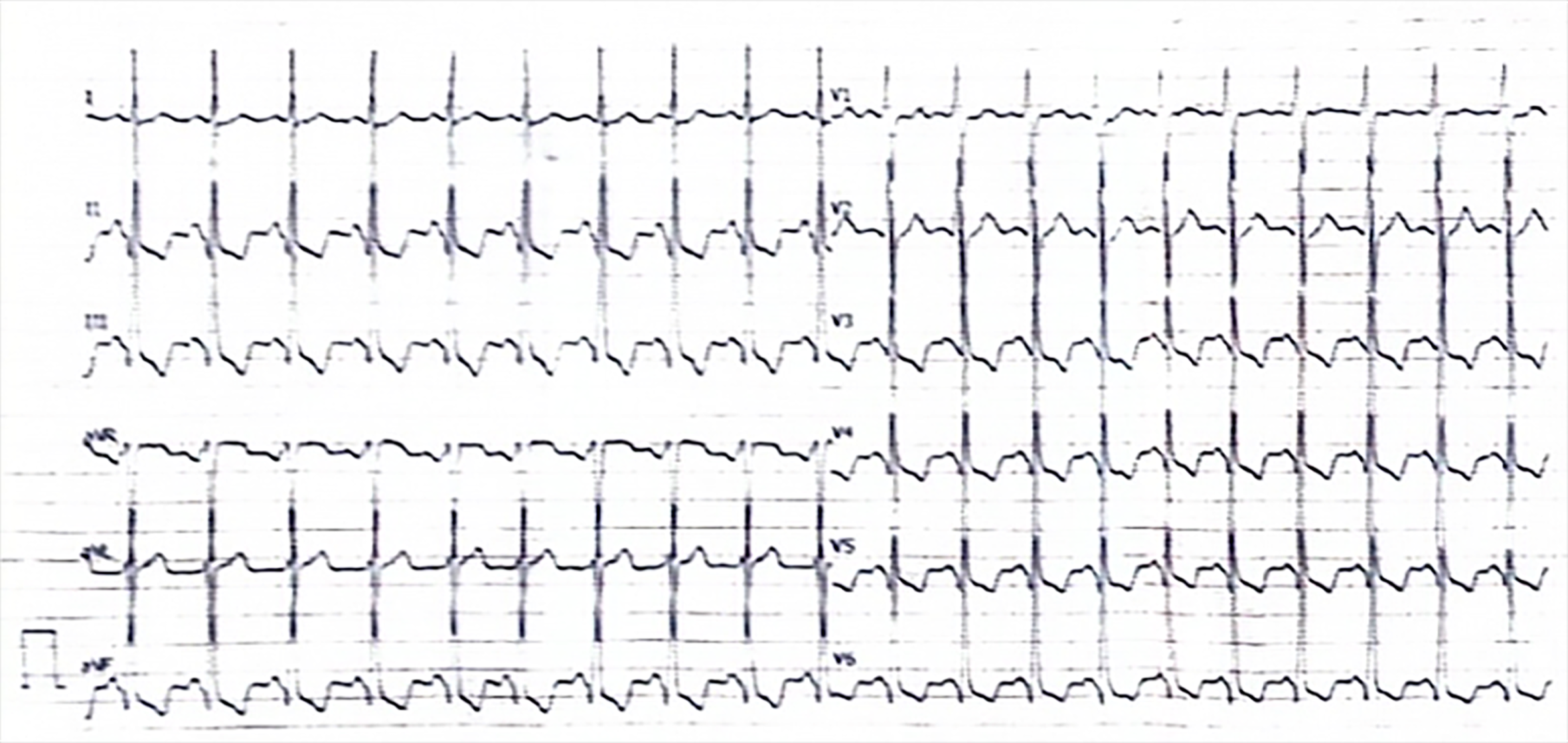

Supplement: Supplementary file 1 [file ijms-25-09171-s001.zip › Figure S1 ECG daughter.tif]

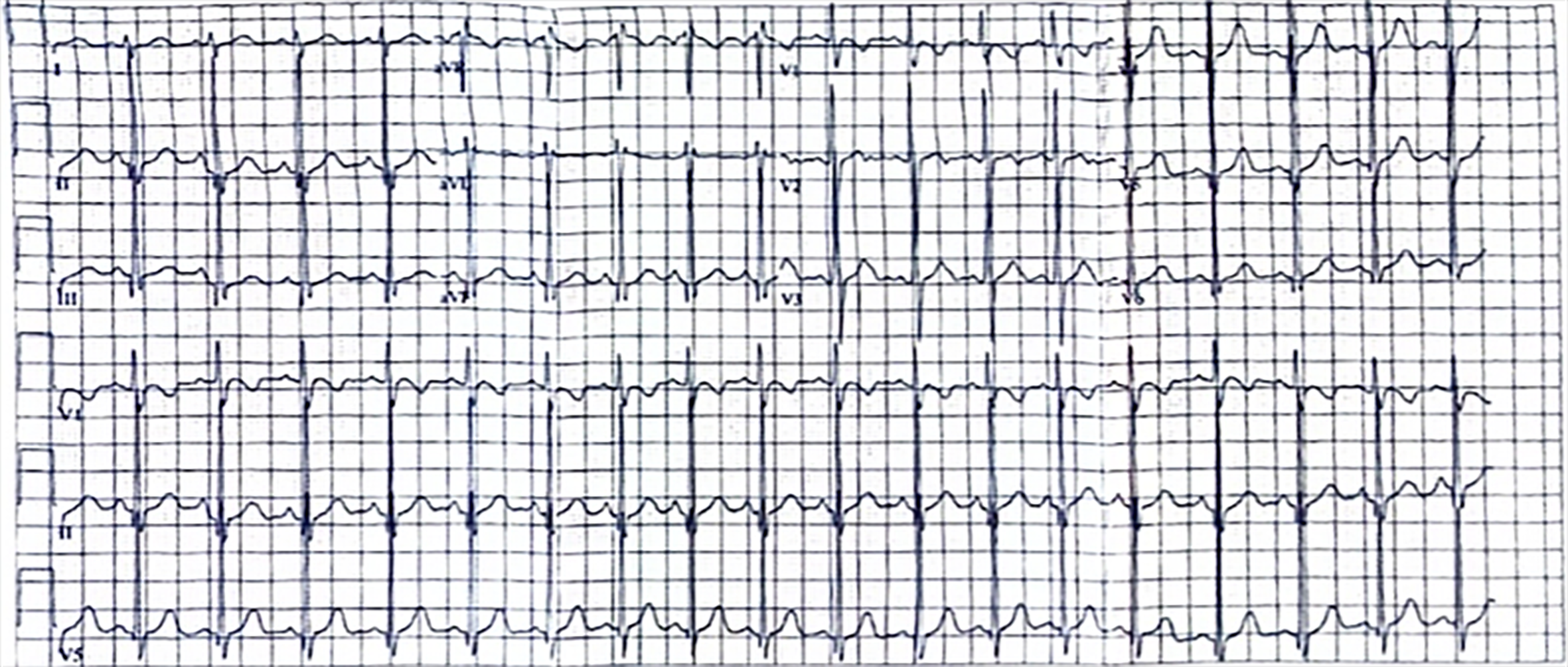

Supplement: Supplementary file 1 [file ijms-25-09171-s001.zip › Figure S2 ECG son.tif]
